# Seroprevalence, spatial clustering, and gender-associated risk factors of porcine cysticercosis in backyard pigs from the high Andean region of Perú: A One Health approach

**DOI:** 10.14202/vetworld.2026.469-480

**Published:** 2026-02-10

**Authors:** Aldo Alim Valderrama Pomé, Martin Equicio Pineda Serruto, William Marcelino Quispe Paredes, Clemente Ochoa Cáceres, Nathaly Velarde Warthon

**Affiliations:** 1 Faculty of Veterinary Medicine and Animal Science, Micaela Bastidas National University of Apurímac, Abancay, Perú; 2 National Institute of Health, Lima, Perú; 3 National Agrarian Health Service of Apurímac, Abancay, Perú; 4 Regional Health Directorate of Apurímac, Abancay, Perú

**Keywords:** backyard pigs, cysticercosis, geographic information system, One Health, Perú, porcine seroprevalence, *Taenia solium*, taeniasis, zoonotic disease

## Abstract

**Background and Ai:**

Porcine cysticercosis caused by *Taenia solium* remains a neglected zoonotic disease in high-altitude Andean regions, where backyard pig-rearing, limited sanitation, and close human–animal–environment interactions promote transmission. Pigs serve as effective sentinels of environmental contamination and offer a practical surveillance proxy for the taeniasis/cysticercosis complex. This study aimed to determine the seroprevalence of porcine cysticercosis, identify associated risk factors, and characterize spatial clustering of infection in backyard pigs in the José María Arguedas district, Apurímac, Perú, using a One Health approach.

**Materials and Methods:**

A quantitative, observational, analytical, cross-sectional study was carried out from April to December 2024. Blood samples were collected from 245 backyard pigs across 49 households and tested for anti-*T. solium* antibodies using the enzyme-linked immunoelectrotransfer blot (EITB) assay. Data on pig and owner characteristics, husbandry practices, sanitation, and knowledge of cysticercosis were gathered through structured questionnaires. Geospatial coordinates of households were recorded and analyzed with geographic information systems (GIS) employing inverse distance weighting interpolation to identify infection hotspots. Associations were assessed using both univariate and multivariate logistic regression analyses.

**Result:**

The overall seroprevalence of porcine cysticercosis was 14.7% (36/245; 95% confidence interval [CI]: 10.1–19.3). Significant spatial clustering was seen, with the Checche locality showing the highest seroprevalence at 60% (p < 0.01), indicating a hyperendemic micro-focus. Univariate analysis revealed associations between porcine cysticercosis and the pig owner’s sex (p < 0.05) as well as awareness that humans can be infected (p < 0.05). Multivariate logistic regression identified only the pig owner’s sex as an independent risk factor, with pigs raised by women having higher odds of infection (Odds ratio = 3.2; 95%CI: 1.3–8.2; p = 0.015). No significant links were found with pig-level characteristics.

**Conclusio:**

This study offers the first combined serological and geospatial assessment of porcine cysticercosis in the José María Arguedas district. The results reveal moderate endemicity, localized transmission hotspots, and an important gender aspect in disease risk. Combining EITB diagnostics with GIS-based mapping within a One Health framework provides a scalable surveillance model to support targeted interventions, health education, especially among women farmers, and sanitation improvements in high-risk Andean areas.

## INTRODUCTION

Cysticercosis is a neglected parasitic disease whose true burden remains significantly underestimated worldwide [1]. Although the disease was identified as potentially eradicable in 1992, it still persists in many endemic regions [2]. Porcine cysticercosis is caused by the cysticercus larvae (metacestodes) of *Taenia solium* [3]. Pigs serve as intermediate hosts and become infected by ingesting eggs or proglottids shed in the feces of humans harboring adult tapeworms. After ingestion, the eggs hatch, migrate through the bloodstream, and encyst in muscle tissues, leading to porcine cysticercosis [2].

This parasitosis has significant public health importance because humans can develop taeniasis after eating undercooked pork contaminated with viable cysticerci, which then mature into adult tapeworms in the small intestine [2, 4]. Additionally, humans may acquire cysticercosis directly through fecal–oral transmission of *T. solium* eggs or proglottids, usually via contaminated food or water. In such cases, cysticerci can lodge in various tissues, including the central nervous system, causing neurocysticercosis, a serious neurological condition often linked to epileptic seizures [1, 2]. Globally, it is estimated that about 2.5 million people are infected with *T. solium*, while nearly 20 million carry cysticerci, resulting in approximately 2.8 million disability-adjusted life years lost each year [2].

From a One Health perspective, the taeniasis/cysticercosis complex involves interactions between human and animal hosts and environmental contamination, especially in developing regions of Asia, Africa, and the Americas [2, 5]. Socioeconomic factors, such as livestock production systems, household income, housing conditions, and food hygiene practices, are crucial to transmission dynamics, as improved sanitation and hygiene are known to reduce the incidence of porcine cysticercosis. In high-altitude Andean communities, human cysticercosis creates significant economic burdens due to diagnostic and treatment costs, reduced productivity, and long-term health problems. Additional losses result from the lower market value of pigs and pork products, or from the complete rejection of carcasses during slaughterhouse inspections [2, 3]. In these areas, pigs are usually raised in extensive, low-input grazing systems and often serve as a form of household savings, which further increases the socioeconomic impact of the disease [6].

Despite the recognized endemicity of *T. solium* taeniasis/cysticercosis across various regions of Perú and other low- and middle-income countries, significant knowledge gaps remain, especially in high-altitude Andean areas. Most existing studies on porcine cysticercosis in Perú have focused on coastal or lowland tropical zones, with limited data from cold, mountainous environments where environmental factors, sanitation infrastructure, and pig-rearing practices differ greatly. Additionally, many previous studies relied on tongue examinations or enzyme-linked immunosorbent assay–based methods, which may underestimate actual exposure compared to the higher specificity of the enzyme-linked immunoelectrotransfer blot (EITB) assay.

Moreover, few studies have combined serological surveillance with geospatial analysis to identify micro-endemic foci and spatial clustering of infection at the household or community level. This integration is crucial for understanding localized transmission patterns and guiding targeted control measures. While socioeconomic and husbandry-related risk factors have been identified, the influence of gender-related factors, especially the sex of pig owners and its impact on knowledge, practices, and infection risk, remains underexplored in Andean backyard production systems. Addressing this gap is vital, as women often play a central role in household pig management in rural communities.

Furthermore, there is a lack of studies explicitly applying a One Health framework that concurrently examines animal infection, human behavior and knowledge, and environmental contamination in endemic rural areas. In the José María Arguedas district of the Apurímac region, no previous seroepidemiological studies have combined EITB diagnostics with geographic information system (GIS) mapping to evaluate porcine cysticercosis. As a result, baseline data on prevalence, spatial distribution, and related risk factors in this high-altitude Andean setting remain limited, hindering evidence-based surveillance, prevention, and control efforts.

Given these gaps, the current study aimed to determine the seroprevalence of porcine cysticercosis in backyard pigs in the José María Arguedas district, Apurímac, Perú, using the EITB assay, a highly specific diagnostic tool. The study also aimed to identify pig- and owner-level factors associated with infection, focusing on socioeconomic characteristics, husbandry practices, sanitation conditions, and knowledge of the taeniasis/cysticercosis complex.

A further objective was to map the spatial distribution of porcine cysticercosis using geospatial analysis of pig-raising households and to identify localized infection hotspots with geographic information system–based methods. By combining serological, epidemiological, and spatial data, this study aimed to implement a One Health surveillance strategy that highlights the interconnected roles of animals, humans, and the environment in disease spread.

Ultimately, the findings of this study aim to provide essential epidemiological data to support targeted, gender-sensitive, and geographically focused control strategies for *T. solium* in high-altitude Andean communities, while contributing to regional and national efforts aligned with the global roadmap for controlling neglected zoonotic diseases.

## MATERIALS AND METHODS

### Ethical approval

The study protocol, which includes the study design, questionnaire, informed consent process, and animal sampling procedures, was reviewed and approved by the Research Ethics Committee of the Apurímac Regional Health Directorate (Approval Minute No. 003, dated July 30, 2024). Before enrollment, all pig owners were thoroughly informed about the objectives, procedures, potential benefits, and minimal risks of the study. Written informed consent was obtained from each participant prior to the collection of epidemiological data and biological samples.

Participation by pig owners was completely voluntary, with no financial rewards or pressure, and participants could withdraw from the study at any time without consequences. Confidentiality was strictly upheld, and household identifiers were anonymized during data analysis and reporting to prevent the recognition of individuals or communities.

All animal handling procedures followed national and international animal welfare standards. The study complied with the Peruvian Animal Protection and Welfare Law (Law No. 30407), which requires humane treatment of animals and the prevention of unnecessary pain or distress during handling and sampling. Additionally, procedures adhered to the recommendations of the World Organization for Animal Health (WOAH) Terrestrial Animal Health Code regarding the welfare of production animals.

Blood sampling was performed by trained veterinarians using standard, minimally invasive techniques designed to minimize stress and discomfort. Proper physical restraint methods were employed, and sampling duration was kept as short as possible. Personnel wore personal protective equipment (PPE) to ensure biosafety and safety for both animals and handlers. Additionally, the study adhered to the Animal Research: Reporting of *In Vivo* Experiments 2.0 guidelines, particularly regarding personnel training, procedural refinement, proper animal handling, and transparent reporting of ethical considerations.

### Study period and location

The study was conducted from April to December 2024 in the rural district of José María Arguedas, Andahuaylas province, Apurímac department, Perú. The district is situated at an average altitude of 3,590 m above sea level, with a mean annual temperature of 13°C and an average annual rainfall of 608.9 mm. Geographically, it is located at latitude 13°44′03″ South and longitude 73°21′02″ West and has a reported poverty rate of 44.6% [7]. The combination of a cool-temperate climate that supports parasite survival and inadequate sanitation infrastructure that leads to environmental contamination creates conditions favorable for the persistence and transmission of cysticercosis, as *T. solium* eggs remain viable for long periods under such conditions.

### Study design

A quantitative, observational, analytical, cross-sectional design was chosen because it enables the simultaneous assessment of multiple exposures and outcomes and is suitable for identifying associations at a single point in time.

### Study population and sample size determination

According to the Fourth National Agricultural Census [8], an estimated 6,201 pigs were raised in backyard production systems within the José María Arguedas district. A simple random sampling method was used, applying the formula:

*n* = NZ²PQ / E²(N−1) + Z²PQ

The minimum sample size was determined based on an expected prevalence of 20% (P), a 5% margin of error (E), and a 95% confidence level (Z), resulting in a needed sample of 237 pigs. Pig-owning households were identified using records from the Integrated Animal Health Management System of the National Agrarian Health Service of Perú [9]. Blood samples were collected from about five pigs per household until the target sample size was achieved. In total, 245 pigs were sampled.

Pigs under 4 months old, pregnant females, animals exhibiting obvious illness, and pigs not raised in backyard systems were excluded from the study.

### Questionnaire design and data collection

Interviewers, who were trained local veterinarians, visited randomly chosen households to gather data through face-to-face interviews conducted in Spanish or, if needed, in the local Quechua language. If no household member was available, the interviewer moved on to the nearest neighboring household.

The questionnaire was validated through expert judgment by a panel of specialists who assessed clarity, relevance, and content validity. The instrument collected information on pig characteristics, owner demographics, pig origin, housing conditions, sanitation practices, feeding systems, slaughter practices, and knowledge related to cysticercosis transmission. Missing or incomplete responses were handled using the missing-at-random (MAR) approach.

### Blood sample collection and serum preparation

Blood samples (6–8 mL) were drawn from the inferior vena cava of pigs using 21G Vacutainer needles and 6 mL Vacutainer tubes without anticoagulant (Botica Labortech company, Perú). Serum was separated by centrifugation at 1000 × *g* for 5 min, frozen, and stored until analysis.

Sampling personnel wore appropriate PPE, including goggles, latex gloves, boots, waterproof coveralls, and masks. Animals were restrained using standard equipment such as mouth gags, stingers, and nose rings to reduce stress and ensure safe sample collection. Samples were transported in insulated containers with cold packs to the Public Health Laboratory of the National University Micaela Bastidas of Apurímac and stored at 2°C–8°C. All samples were labeled and tracked through a documented chain-of-custody process.

### Serological diagnosis using the EITB assay

Serological detection of porcine cysticercosis was conducted using the EITB assay to identify antibodies against *T. solium*. Samples were considered positive if they showed reactivity against at least one of seven lentil lectin–purified metacestode glycoproteins (GP; GP50, GP42–39, GP24, GP21, GP18, GP14, and GP13 kDa) [10, 11].

Among the positive samples, 26 showed a single reactive band, 7 showed two bands, and 3 showed three bands. All assays were performed at the Parasitic Immunology Laboratory of the Universidad Peruana Cayetano Heredia (Lima, Perú) following standardized protocols [10, 11]. The EITB assay detects exposure to *T. solium* eggs and both viable or past infections, and it is considered the reference method for population-level surveillance [6, 12].

### Quality control of the EITB assay

Quality control procedures highlighted the high sensitivity and specificity of the EITB assay. The positive control consistently exhibited the characteristic seven-band pattern associated with confirmed cysticercosis, confirming antigen integrity and reagent performance. Negative control sera showed no band reactivity.

To ensure specificity, sera from individuals with other parasitic infections were used to evaluate potential cross-reactivity. Samples with weak or atypical banding patterns were retested. The assay employs lentil lectin affinity–purified GPs derived from *T. solium* cyst fluid, originally developed by the U. S. Centers for Disease Control and Prevention, with stable reagents prepared under controlled laboratory conditions [10, 11].

### Geospatial mapping and hotspot identification

The spatial distribution of porcine cysticercosis was examined using GIS techniques. A total of 49 pig-raising households across 10 localities were georeferenced with a GARMIN eTrex® 10 GPS device, with an accuracy of <10 m. Coordinates were processed in RStudio (R version 4.4.1, R Core Team, Vienna, Austria) utilizing the raster and tidyverse packages.

Inverse Distance Weighting interpolation was used to visualize spatial clustering in accordance with Tobler’s first law of geography. This method gives greater weight to nearby points when estimating values at unsampled locations, creating a continuous raster surface. Infection foci were defined as areas with more than two seropositive households within a 200 m radius.

### Statistical analysis

All collected data were checked for completeness, consistency, and accuracy before analysis. Data cleaning involved identifying and correcting entry errors, resolving inconsistencies, and managing missing values. Missing or incomplete responses were addressed using the MAR assumption to reduce potential bias. Qualitative variables were coded into standardized numerical formats to facilitate statistical processing.

The cleaned dataset was entered into Microsoft Office Excel 365 (Microsoft Corp., Washington, USA) for initial organization and then analyzed using R statistical software version 4.4.1 (R Core Team, Vienna, Austria). Descriptive statistics summarized pig owners’ characteristics, pig demographics, husbandry practices, and sanitation variables. Categorical variables were presented as frequencies and percentages.

Associations between porcine cysticercosis seropositivity and categorical explanatory variables were first evaluated using chi-square tests. Crude odds ratios (ORs) with 95% confidence intervals (CIs) were then calculated to measure the strength of these associations. Variables with p-values ≤ 0.05 in bivariate analyses were considered statistically significant and were included in multivariate modeling.

Univariate logistic regression analyses were performed to identify potential risk factors associated with pig owners’ characteristics (such as sex, education level, knowledge of cysticercosis transmission, pork consumption habits, latrine ownership, pig confinement, access to latrines, number of pigs reared, feed type, and home slaughter practices) and pig-related factors (including sex, reproductive class, origin, and housing location).

A multivariate logistic regression model was then developed to identify independent predictors of porcine cysticercosis while controlling for confounding variables. Backward stepwise elimination based on the Wald statistic was used, starting with a full model that included all candidate variables. Variables with p-values > 0.05 were sequentially removed, and adjusted ORs with 95% CIs were reported for the final model. Statistical significance was set at p ≤ 0.05.

Potential confounding was managed both during the design phase (using standardized inclusion and exclusion criteria) and during the analysis phase (via multivariate adjustment). Model assumptions were checked, and the findings were interpreted in light of the cross-sectional design. It is recognized that the identified associations and spatial risk patterns might not be directly applicable to regions with different environmental conditions or pig production systems.

## RESULTS

### Characteristics of pig farmers

All pig farmers reported having access to treated water (100%), and the average age of respondents was 40.5 years (standard deviation [σ] = 13.5). As shown in Table 1, most pig owners were women (67.3%) and had completed primary education (40.8%). The majority of respondents reported consuming pork products quarterly (85.1%). Sanitation coverage was high, with 93.9% of households possessing latrines, and 91.8% allowing pigs access to these facilities. Regarding husbandry practices, most farmers raised fewer than 10 pigs (93.9%) and primarily fed them household waste (89.8%).

**Table 1:** Characteristics of pig farmers in the José María Arguedas district, Andahuaylas, Apurímac, Perú, 2024.

Characteristics	n	%
Sex		
Female	165	67.3
Male	80	32.7
Education level		
High school	90	36.7
Primary	100	40.8
No schooling	55	22.4
Knowledge		
Knows what cysticercosis is and	95	38.8
Knows that pigs can become infected	120	49.0
Knows that people can get infected	100	40.8
Consumption of pork products		
Quarterly	200	85.1
Monthly	35	14.9
Has a latrine	230	93.9
Keeps confined pigs	15	6.1
Access to the latrine	225	91.8
Number of raised pigs		
≤10	230	93.9
>10	15	6.1
Feed given to pigs		
Balanced	5	2.0
Waste	220	89.8
Mixed	20	8.2
Performs the home slaughter of animals	45	19.6

### Characteristics of pigs raised in backyard systems

The demographic characteristics of pigs raised in backyard systems are shown in Table 2. Most pigs were females (56.3%), classified as adults (64.1%), and mainly obtained from livestock fairs (69.4%).

**Table 2:** Characteristics of pigs raised in the José María Arguedas district, Andahuaylas, Apurímac, Perú, 2024.

Characteristics	n	%
Sex of the pigs		
Female	138	56.3
Male	107	43.7
Reproductive class		
Piglet	23	9.4
Young	65	26.5
Adult	157	64.1
Origin of pigs		
Fair	170	69.4
Neighbors	70	28.6
Mixed	5	2.0

### Overall seroprevalence of porcine cysticercosis

The overall seroprevalence of porcine cysticercosis was 14.7% (36/245; 95% CI = 10.1–19.3). As shown in Table 3, no statistically significant differences were observed in the prevalence of cysticercosis based on the location where pigs were obtained (p > 0.05).

**Table 3:** Seroprevalence of cysticercosis according to the locality of origin of pigs kept in the José María Arguedas district, Andahuaylas, Apurímac, Perú, in 2024.

Locality	Pigs with cysticercosis n (%)	Pigs without cysticercosis n (%)	Total (100%)	p-value
Andahuaylas	26 (15.3)	144 (84.7)	170	0.309
Checche	2 (40.0)	3 (60.0)	5	0.448
Cumanaylla	3 (10.0)	27 (90.0)	30	0.999
Cruz Pampa	1 (20.0)	4 (80.0)	5	0.999
Cruz Pampa-Huancabamba	1 (5.0)	19 (95.0)	20	0.999
Huancabamba	2 (40.0)	3 (60.0)	5	0.999
Lumanaylla	0 (0.0)	5 (100.0)	5	0.999
Ñahuimpuquio	1 (20.0)	4 (80.0)	5	0.999

### Spatial distribution and locality-level prevalence

**Figure 1:**
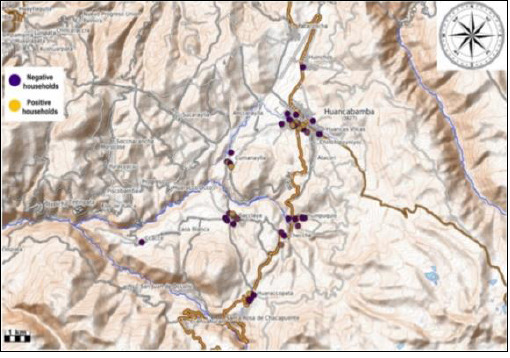
Distribution map of houses from which serological samples from pigs with (yellow) and without (purple) cysticercosis were obtained in localities of the José María Arguedas district, Andahuaylas, Apurímac, Perú, in 2024.

**Figure 2:**
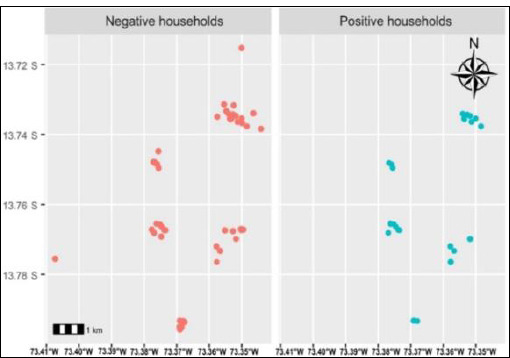
Georeferencing of the distribution of households with serological samples from pigs with cysticercosis (in light blue) and without cysticercosis (in pink), in localities of the José María Arguedas district, Andahuaylas, Apurímac, Perú, in 2024.

A total of 10 localities within the José María Arguedas district were sampled, with approximately five pigs examined per household. Table 4 shows that the Checche locality had the highest seroprevalence of porcine cysticercosis (60%; 95% CI = 26–87), which was significantly greater than that of other localities (p < 0.05). This spatial clustering is depicted in the serological sample distribution map (Figure 1) and the georeferenced household-level distribution of pigs with and without cysticercosis across the district (Figure 2).

**Table 4:** Seroprevalence of cysticercosis according to the localities where pigs were kept in the José María Arguedas district, Andahuaylas, Apurímac, Perú, in 2024.

Locality	Pigs with cysticercosis n (%)	Pigs without cysticercosis n (%)	Total (100%)	p-value
Checche	6 (60.0)	4 (40.0)	10	**0.009
Cumanaylla	3 (10.0)	27 (90.0)	30	0.999
Cruz Pampa	2 (8.0)	23 (92.0)	25	0.999
Huancabamba	9 (16.4)	46 (83.6)	55	0.999
Huaroccopata	4 (11.4)	31 (88.6)	35	0.999
Huinchos	0 (0.0)	5 (100.0)	5	0.999
Lumanaylla	0 (0.0)	5 (100.0)	5	0.999
Ñahuimpuquio	4 (11.4)	31 (88.6)	35	1.000
Sacclaya	8 (20.0)	32 (80.0)	40	0.999

** p < 0.01

### Risk factor analysis: univariate and multivariate models

Univariate logistic regression analysis showed that the sex of the pig owner (18.2%; 95% CI = 11.9–24.4; p < 0.05) and awareness that humans can be infected with cysticercosis (p = 0.021; 95% CI = 12.5–29.5) were significantly linked to porcine cysticercosis (Table 5).

**Table 5:** Characteristics of pig owners with cysticercosis in the José María Arguedas district, Andahuaylas, Apurímac, Perú, 2024.

Characteristics	Pigs with cysticercosis n (%)	Pigs without cysticercosis n (%)	Total (100%)	p-value
Sex				
Female	30 (18.2)	135 (81.8)	165	*0.027
Male	6 (7.5)	74 (92.5)	80	
Education level				
High school	12 (13.3)	78 (86.7)	90	
Primary	16 (16.0)	84 (84.0)	100	0.874
No schooling	8 (14.5)	47 (85.5)	55	
Knowledge				
Knows what cysticercosis is	14 (14.7)	81 (85.3)	95	0.988
Knows pigs can become infected	22 (18.3)	98 (81.7)	120	0.115
Knows people can get infected	21 (21.0)	79 (79.0)	100	*0.021
Consumption of pork products				
Quarterly	27 (13.5)	173 (86.5)	200	0.738
Monthly	4 (11.4)	31 (88.6)	35	
Sanitation and management				
Has a latrine	34 (14.8)	196 (85.2)	230	0.878
Keeps confined pigs	1 (6.7)	14 (93.3)	15	0.365
Access to latrine	4 (20.0)	16 (80.0)	20	0.484
Number of raised pigs				
≤ 10	34 (14.8)	196 (85.2)	230	0.878
> 10	2 (13.3)	13 (86.7)	15	
Feed given to pigs				
Balanced	1 (20.0)	4 (80.0)	5	0.787
Waste	33 (15.0)	187 (85.0)	220	
Mixed	2 (10.0)	18 (90.0)	20	
Home slaughter of animals	3 (6.7)	42 (93.3)	45	0.075

* p < 0.05

No statistically significant links were found between pig-related traits and the presence of porcine cysticercosis (p > 0.05) (Table 6).

**Table 6:** Characteristics of porcine cysticercosis in pigs raised in the José María Arguedas district, Andahuaylas, Apurímac, Perú, in 2024.

Characteristics	Pigs with cysticercosis n (%)	Pigs without cysticercosis n (%)	Total (100%)	p-value
Sex of the pigs				
Female	17 (12.3)	121 (87.7)	138	0.233
Male	19 (17.8)	88 (82.2)	107	
Reproductive class				
Piglet	6 (26.1)	17 (73.9)	23	0.078
Young	5 (7.7)	60 (92.3)	65	
Adult	25 (15.9)	132 (84.1)	157	
Origin of pigs				
Fair	26 (15.3)	144 (84.7)	170	0.840
Neighbors	1 (20.0)	4 (80.0)	5	
Mixed	9 (12.9)	61 (87.1)	70	

In the multivariate logistic regression analysis, only the sex of the pig owner remained independently associated with porcine cysticercosis. Pigs raised by female owners had a significantly higher likelihood of infection (OR = 3.2; 95% CI = 1.3–8.2; p = 0.015) (Table 7).

**Table 7:** Multivariate logistic regression analysis (backward: wald).

Characteristic^a^	B	Standard error	Wald	df	Significance	Exp(B)	95% CI for Exp(B) (Lower)	95% CI for Exp(B) (Upper)
Woman	1.162	0.479	5.892	1	*0.015	3.198	1.251	8.176
Piglet	–	–	6.219	2	*0.045	–	–	–
Young	0.739	0.539	1.883	1	0.170	2.094	0.729	6.018
Adult	−0.947	0.519	3.325	1	0.068	0.388	0.140	1.073
Constant	−2.518	0.448	31.558	1	0.000	0.081	–	–

a Specified variables: sex and productive class.

* p < 0.05, CI = Confidence interval.

## DISCUSSION

### Seroprevalence and epidemiological context

The overall seroprevalence of porcine cysticercosis observed in this study was 14.7%, confirming that this parasitic infection remains endemic in the José María Arguedas district. The persistence of porcine cysticercosis poses a major public health concern and causes economic losses for rural communities, mainly through reduced market value or condemnation of pig carcasses [13]. The prevalence reported here is similar to figures documented in Callao, Perú [14], and in several endemic countries, including Gambia and Senegal [1], Burma [15], Ivory Coast [16], and Tanzania [17–19]. However, higher prevalences have been reported in nearby Andean localities such as Nueva Esperanza, Turpo, and Matapuquio [3], as well as in other Peruvian regions, including Tumbes [13, 20, 21], Amazonas [22], and Tarapoto [23]. Likewise, elevated prevalences have been recorded in Ecuador [24], Colombia [25], Guatemala [26], South Africa [27], Zambia [28], Tanzania [29], Kenya [30], Cameroon [31], Ghana [32], and Indonesia [33], indicating that the level observed in this study reflects moderate endemicity.

Unlike tropical lowland settings, high-altitude Andean backyard production systems are characterized by distinctive ecological and management conditions, including limited waste-disposal infrastructure and climatic factors that may prolong the survival of *T. solium* eggs in the environment, thereby affecting transmission dynamics.

### Sanitation, husbandry practices, and environmental contamination

Access to human feces, latrine availability, and pig confinement are well-known risk factors for porcine cysticercosis [22, 34–36]. In this study, no significant links were found between porcine cysticercosis and pig confinement or latrine ownership. This may be due to the poor quality of most latrines, which were often basic and lacked secure barriers, allowing pigs to access them. Given pigs’ coprophagous behavior and the dominance of free-ranging management systems in the district, exposure to human feces is likely to persist despite limited sanitation coverage.

Furthermore, the widespread practice of feeding pigs with household waste (89.8%) may increase exposure to *T. solium* eggs [14]. Environmental contamination can be worsened by poor personal hygiene and the presence of untreated human tapeworm carriers. Additionally, water sources may become contaminated through open defecation, which can help spread the eggs, as *T. solium* eggs can survive in both stagnant and flowing water for long periods [29].

### Spatial heterogeneity and hyperendemic foci

Although no differences were observed in seroprevalence based on the locality where pigs were acquired, significant spatial heterogeneity was evident at the household-level. The Checche locality showed a notably high seroprevalence (60%), indicating a hyperendemic focus. This high prevalence is likely related to the remoteness of Checche and its limited sanitation infrastructure, factors known to hinder livestock commercialization and increase the risk of animal neglect [3, 37].

Cold and humid Andean soils may further support egg survival, maintaining environmental reservoirs even in areas with relatively low human infection pressure. Additionally, the practice of home slaughter, reported by nearly one-fifth of pig owners, increases the risk of human exposure to infected meat, emphasizing the importance of pre-slaughter inspection [29]. The observed spatial clustering underscores the value of GIS–based analyses for identifying micro-endemic foci and prioritizing targeted interventions.

### Knowledge-related factors and behavioral determinants

Knowledge that humans can become infected with cysticercosis was significantly linked to porcine cysticercosis, consistent with findings from Tanzania, where limited awareness among household members was associated with increased pig seropositivity [18, 38, 39]. Previous studies have also indicated connections between prior herd infection and ongoing transmission [29]. Notably, Puerta *et al*. [34] reported that only 15% of pig farmers knew the routes of human infection.

Limited understanding of cysticercosis and its transmission cycle can lead to practices that sustain the parasite’s life cycle [40–42]. These results highlight the importance of ongoing health education programs focused on the life cycle of *T. solium*, zoonotic risks, and related economic impacts, as lack of knowledge can hinder control and eradication efforts [25, 32, 43].

### Gender-related risk and implications for control strategies

A key finding of this study was the link between the sex of pig owners and porcine cysticercosis, with pigs raised by women showing a threefold higher risk of infection (OR = 3.2). This aligns with prior reports indicating that women have less cysticercosis-related knowledge compared to men [43]. In rural areas, men often travel to urban centers for work, gaining more exposure to health information, while women, mainly involved in household and animal care, may have fewer opportunities to access such knowledge.

These results highlight the need to include a gender-sensitive approach in cysticercosis control programs. Intersectoral and geotargeted strategies are recommended, using GIS-based hotspot identification to inform surveillance and quick response [3]. Control efforts should focus on targeted health education for women pig farmers, improving sanitation infrastructure and waste management in high-risk areas, and enhancing animal health interventions, such as deworming and meat inspection, all within a comprehensive One Health framework.

## CONCLUSION

This study showed that porcine cysticercosis remains endemic in the high-altitude district of José María Arguedas, Apurímac, Perú, with an overall seroprevalence of 14.7% as determined by the ETIB assay. Spatial analysis uncovered significant variation in transmission, with the Checche locality identified as a hyperendemic micro-focus (60%), highlighting localized environmental and sanitation-related factors driving infection. Among the evaluated risk factors, the sex of the pig owner was the only independent predictor of porcine cysticercosis, with pigs raised by female owners having a threefold higher risk of infection. No significant links were found with pig-level characteristics, reinforcing the primary role of human behavior and environmental contamination in transmission dynamics.

These findings highlight the importance of pigs as effective biosentinels for monitoring environmental contamination with *T. solium* eggs, especially in rural Andean areas where human surveillance is expensive and logistically difficult. Combining EITB diagnostics with geographic information system–based mapping offers a practical, scalable One Health surveillance tool for detecting micro-endemic hotspots and guiding targeted interventions. Health education programs should be geographically focused and gender-sensitive, with special attention to women pig farmers, sanitation improvements, safe latrine design, and awareness of zoonotic transmission. Improving meat inspection practices and discouraging unregulated home slaughter are also crucial to reduce human exposure.

A key strength of this study is the use of the EITB assay, which provides high specificity and reliability for population-level seroepidemiological surveillance. Combining serological data with geospatial analysis enabled precise identification of localized hotspots, going beyond simple district-level prevalence estimates. Additionally, the study applied a One Health framework by simultaneously evaluating animal infections, human knowledge and practices, and environmental factors in a high-altitude Andean setting that is rarely represented in the literature.

The cross-sectional design limits the ability to draw causal links between identified risk factors and infection status. Human taeniasis was not directly measured, which prevents confirming active environmental conta-mination sources. Additionally, pigs are often sold or slaughtered for cultural or economic reasons, restricting the ability to perform longitudinal follow-up or measure incidence. Lastly, the results may not be directly applicable to regions with different ecological conditions or pig production systems.

Future studies should use longitudinal or intervention-based designs to assess the effects of targeted sanitation improvements, gender-focused education, and integrated human–animal treatment strategies. Simultaneous evaluation of human taeniasis, environmental contamination, and pig infection would give a more comprehensive understanding of transmission dynamics. Extending GIS-based surveillance to other Andean districts could aid regional risk mapping and support national control programs aligned with the World Health Organization’s roadmap for neglected zoonotic diseases.

In conclusion, porcine cysticercosis remains a persistent One Health challenge in high-altitude Andean communities. By combining highly specific diagnostics with spatial analysis, this study provides actionable evidence to support geotargeted, gender-sensitive, and integrated control strategies. These approaches are essential for reducing transmission, minimizing economic losses, and progressing toward the sustainable control of *T. solium* in endemic rural settings.

## DATA AVAILABILITY

The supplementary data can be made available from the corresponding author upon request.

## AUTHORS’ CONTRIBUTIONS

AV and MP: Conceived, designed, and coordinated the study and developed the data collection tools. AV, MP, and CO: Supervised field sampling, data collection, laboratory work, and data entry. AV: Managed the project, performed statistical analysis and interpretation, wrote the manuscript, and created visualizations. AV, WQ, and NV: Coordinated the study, conducted critical reviews of the manuscript, and ensured compliance with ethical protocols. All authors have read and approved the final version of the manuscript.

## COMPETING INTERESTS

The authors declare that they have no competing interests.

## PUBLISHER’S NOTE

Veterinary World remains neutral with regard to jurisdictional claims in the published institutional affiliations.
